# Case report: Case report and literature review: Treatment of sweat gland carcinoma

**DOI:** 10.3389/fonc.2023.1091934

**Published:** 2023-01-23

**Authors:** Yuhong Dai, Jin Feng, Xiao Zhou

**Affiliations:** Department of Oncology, Tongji Hospital, Tongji Medical College, Huazhong University of Science and Technology, Wuhan, China

**Keywords:** sweat gland carcinoma, surgery, radiotherapy, chemotherapy, immunotherapy

## Abstract

**Background:**

Sweat gland carcinoma (SGC) is a rare neoplasm originating from sweat glands. Surgical resection is the first choice for SGC treatment, and there is no consensus on other treatments for advanced SGC.

**Methods and result:**

In this case report, we present a case of a female patient with advanced SGC who received surgery; radiotherapy; multiple lines of chemotherapy, which include docetaxel, nedaplatin, albumin-bound paclitaxel, and pemetrexed; and immunotherapy (camrelizumab). The survival time of this patient is 35 months. MRI tumor monitoring indicated that these treatments slowed the progression of the disease. The effectiveness of chemotherapy, radiotherapy, and immunotherapy should be tested for more patients with SGC in the future.

**Conclusion:**

Although the patient’s tumor was uncontrolled eventually, multiple treatments delayed tumor growth over a period of time, providing ideas for others when choosing regimens.

## Introduction

Sweat gland carcinoma (SGC) is a rare type of skin cancer originating from sweat glands. The incidence rate is less than 0.001% of all tumors (1). SGC usually occurs on the head, neck, and the lower extremities in patients between 60 and 70 years of age, with no gender preference (2). Most cases present as mass and nodule in the primary site. It also has an aggressive characteristic with potential for nearby lymph node invasion and distant metastasis (3). Generally, surgical resection is the first choice for SGC treatment. The therapeutic effect of radiotherapy, chemotherapy, and immunotherapy has not been confirmed. Due to the limited research on this disease, diagnosis and therapy remain to be a challenge. Herein, we present a female patient with advanced SGC who received surgery, multiple lines of chemotherapy, immunotherapy, and radiotherapy with temporary benefit.

## Case presentation

The patient was a 55-year-old woman who had a small itchy nodule in the left waist 20 years ago and received cryotherapy twice in 2006 and 2014. However, she still felt itchy and the nodule continued to grow. Then, she came to our hospital for further diagnosis and treatment.

**Figure 1:**
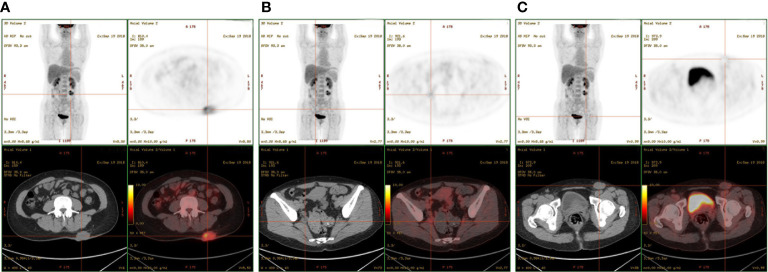
PET-CT showed multiple masses located in the **(A)** left lower back, **(B)** right pelvic wall, and **(C)** inguinal region.

**Figure 2:**
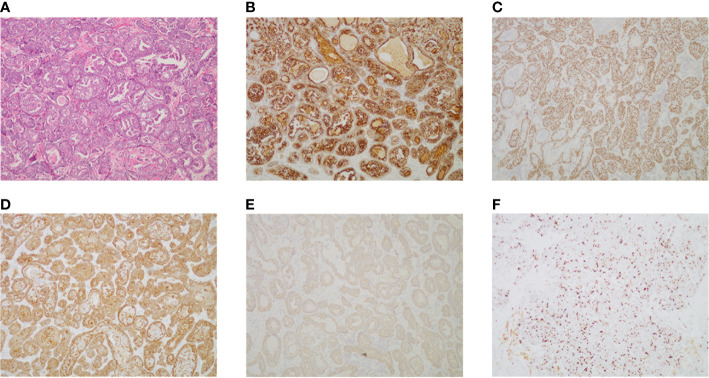
**(A)** Pathologic examination of tumor tissue confirmed typical SGC histological features (hematoxylin and eosin stain 100). **(B)** The tumor cells were positive for CK7 (CK7 stain 100). **(C)** The tumor cells were positive for P63 (P63 stain 100). **(D)** The tumor cells were positive for CK5/6 (CK5/6 stain 100). **(E)** The tumor cells were positive for partial CK14 (CK14 stain 100). **(F)** The expression of Ki-67 in tumor tissue (Ki-67 stain 100).

**Figure 3:**
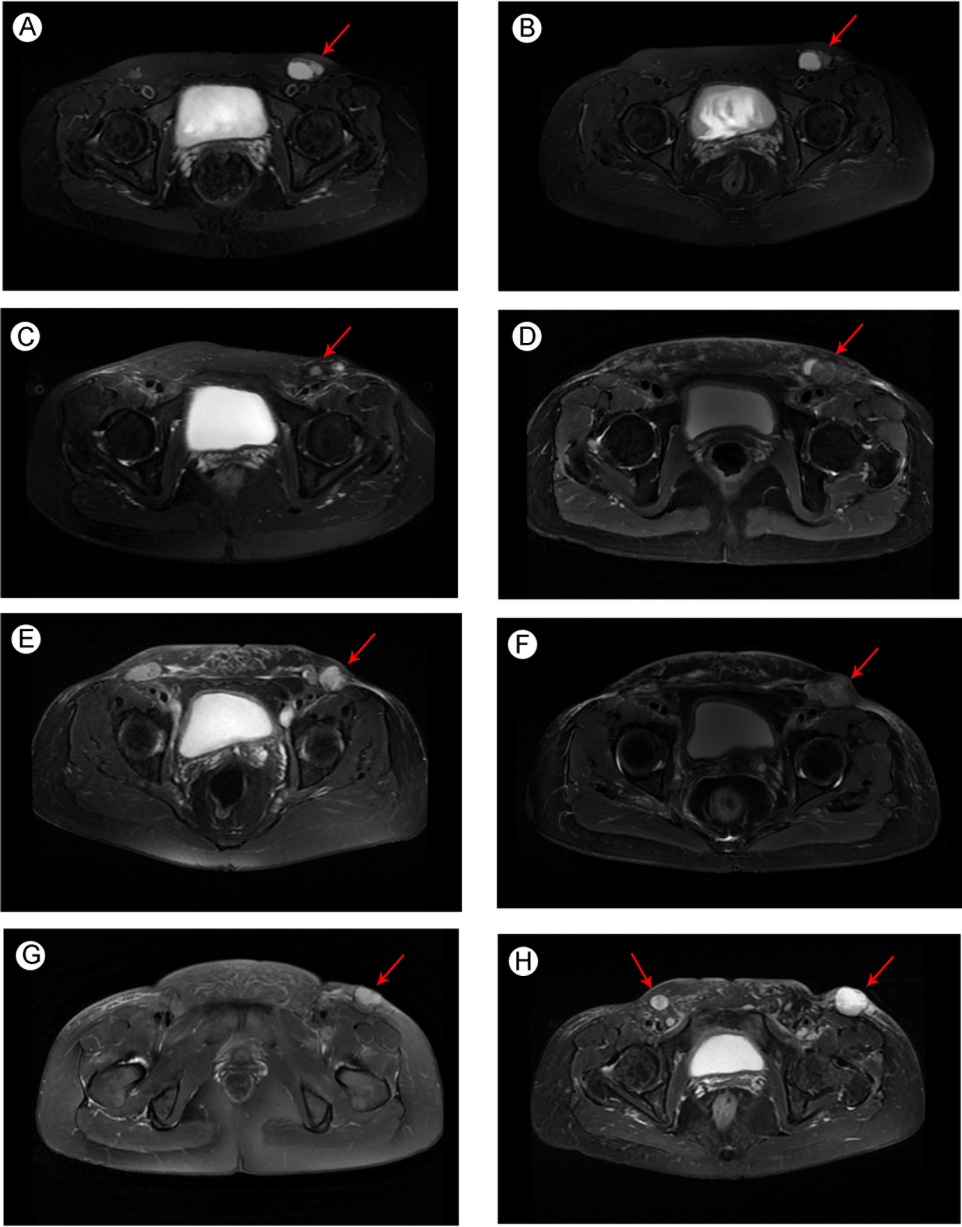
Pelvic MRI image of metastatic SGC **(A)** before docetaxel + nedaplatin treatment in October 2018; **(B)** after docetaxel + nedaplatin treatment in February 2019; **(C)** after the radiotherapy in September 2019; **(D)** before albumin-bound paclitaxel + camrelizumab treatment in December 2019; **(E)** after albumin-bound paclitaxel + camrelizumab treatment in September 2020; **(F)** before pemetrexed + nedaplatin + camrelizumab treatment in December 2020; and newly developed metastatic lymph nodes **(G)** before pemetrexed + nedaplatin + camrelizumab treatment in December 2020; **(H)** after pemetrexed + nedaplatin + camrelizumab treatment in March 2021. Arrows indicate the tumors in the groin region.

**Figure 4:**
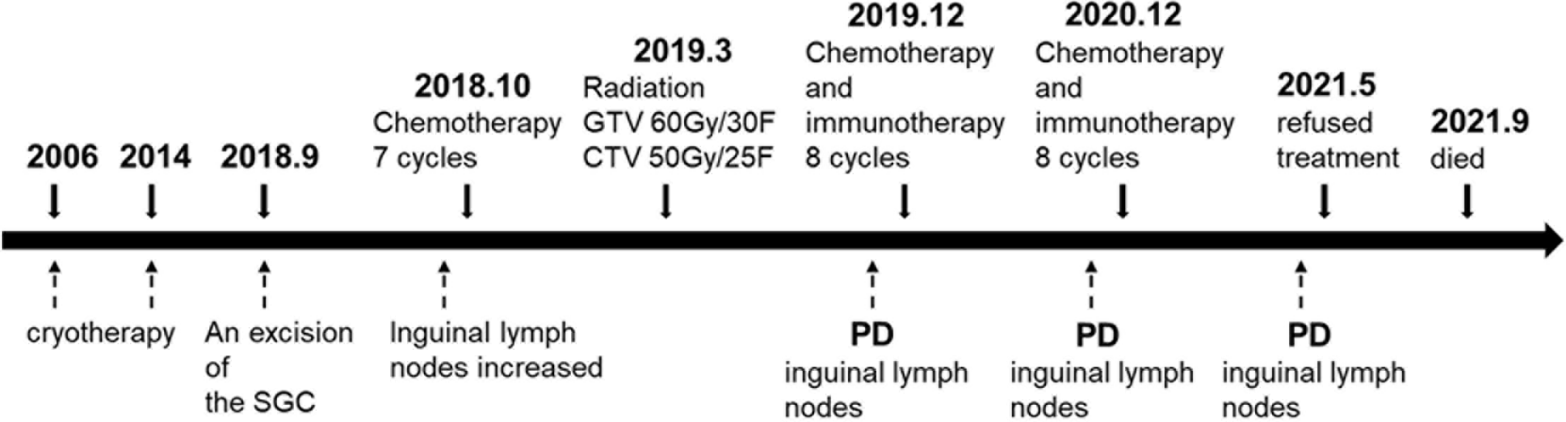
Summary of the treatment timeline of this case.

The patient had no previous medical history and no notable personal and family history. Physical examination revealed a palpable mass of about 4 × 2 cm in the left lower back and enlarged lymph nodes in the left groin. Laboratory tests showed that the complete blood count and blood biochemical indicators were basically within the normal range. A positron emission tomography/computed tomography (PET/CT) scan was done on 21 September 2018, and it revealed a malignant tumor that was located on the left lower back with right pelvic wall and bilateral inguinal lymph node metastasis (**Figures 1A-C**). Surgery was performed to remove the lesion on the left lower back on 25 September 2018 with a tumor-free margin. Pathological inspections confirmed the diagnosis of SGC, and immunohistochemistry results indicated that the mass was CK7+, P63+, CK5/6+, CK14+partial, GCDFP-15−, GATA-3−, p40−, and Ki-67 10% (**Figures 2A-F**). Her magnetic resonance (MR) scanning image on 12 October 2018 showed that the number of lymph nodes increased in the bilateral inguinal region, and the left one was enlarged, suggestive of malignant disease (**Figure 3A**). Because the left lesions were surgically unresectable, the patient agreed to undergo a cytotoxic chemotherapy regimen consisting of docetaxel and nedaplatin every 3 weeks for seven cycles. MRI revealed stabilization of the disease (**Figure 3B**). Then, she received intensity-modulated radiation therapy (IMRT) with 60 Gy to the gross tumor volume (GTV) and 50 Gy to the clinical tumor volume (CTV). After the radiotherapy, the patient was reviewed regularly, and the tumor size was obviously reduced (**Figure 3C**). However, MR scanning on 4 December 2019 revealed that the inguinal lymph nodes became larger (**Figure 3D**). We recommended that she undergo whole-genome sequencing. Due to the high price and limited specimen, only peripheral blood was used to detect PD-L1 expression. PD-L1 expression was 10%. Then, she underwent a chemotherapy and immunotherapy regimen consisting of albumin-bound paclitaxel and camrelizumab (a PD-1 inhibitor) for eight cycles. During the treatment, MRI showed stabilization of the disease (**Figure 3E**). On 14 December 2020, MR scanning showed that the number and size of the inguinal lymph nodes increased and were larger than the previous ones (**Figures 3F, G**). We repeatedly communicated with the patient about the treatment plan and told her that the prognosis was not good, but the patient still agreed to continue the treatment. Then, the patient underwent chemotherapy and immunotherapy with pemetrexed, nedaplatin, and camrelizumab for eight cycles. However, the inguinal lymph nodes were poorly controlled and growing larger according to the MRI result (**Figure 3H**). Then, the patient refused further treatment and went back home. Through telephone follow-up, we learned that the tumors in the left groin continued to grow and were ulcerating. The patient gradually developed pleural fluid and ascites. She died on 2 September 2021. To date, the survival time of this patient is 35 months. The timeline for treatments and efficacy evaluations in this case is summarized in **Figure 4**.

## Discussion

Due to the limited available data in the literature and the wide variety of clinical symptoms, the diagnosis and treatment of SGC are very complex and difficult. There is no consensus on the diagnosis and treatment of this disease.

The first case of SGC was reported in 1895 by a pathologist, V. Cornil (4). SGC was classified according to histopathological type in 1968 by Berg (5). According to the 2018 WHO classification criteria, the pathological classification of the SGC is very complex and can be subdivided into more than 10 types, such as adnexal adenocarcinoma, porocarcinoma, mucinous carcinoma, and syringocystadenocarcinoma papilliferum, among others. In traditional views, since the biological behavior of this disease is unpredictable, the diagnosis is mainly based on immunohistochemistry rather than clinical appearance. SGC is usually divided into apocrine gland carcinomas and eccrine carcinomas. Apocrine gland carcinomas present as non-tender single or multiple, firm, rubbery, or cystic masses overlying skin (6, 7). Microscopically, they are characterized by adenocarcinomas with well-developed glandular lumina. Tumor cells stained positive for PAS since they contain glycogen granules and they are diastase resistant (8). Eccrine carcinomas have no specific clinical features, making it very difficult to diagnose by gross appearance. They usually manifest as non-tender, subcutaneous nodules. The microscopic structures show a narrow lumen surrounded by a layer of flattened or cuboidal cells without hemosiderin. Tumor cells stain positive for PAS and are diastase sensitive (7). However, some researchers believed that it is difficult to separate the benign neoplasms and cutaneous metastases of visceral primary adenocarcinomas based on immunohistochemistry (9). They separate SGC into low- and high-grade malignant neoplasms according to their clinical behavior. Low-grade malignant tumors could grow destructively and recur in the local region, leading to significant morbidity. High-grade malignant tumors can easily metastasize to distant organs and show significant disease-related mortality (9).

SGC is distributed equally in male and female patients, with a peak incidence in the seventh and eighth decades of life (10). The most commonly affected primary sites are the head and neck, followed by the lower extremities. The most common organs of metastasis are the regional lymph nodes, followed by the lungs (2). Usually, SGC is converted from a benign subcutaneous mass. The mean duration of presentation was 5.57 years, ranging from 4 days to 60 years (2).

The recommended treatment for SGC is wide surgical excision. Recent studies show that Mohs micrographic surgery is the preferred option compared to conventional surgical excision (11). In Thomas’s study, a retrospective analysis of 26,000 cases confirmed that Mohs micrographic surgery is the most effective therapy for aggressive cutaneous malignancies, which can significantly reduce the recurrence rate (12). The therapeutic effect of radiotherapy on SGC has not been confirmed. SGC was previously believed to be radio-resistant in earlier reports (13). In recent studies, radiotherapy is commonly used in postoperative adjuvant therapy at a dose of 50–70 Gy with a good control of local recurrence (14). However, the efficacy of radiotherapy is still controversial, and more studies are needed to help us understand the role of radiotherapy in SGC. The role of chemotherapy in SGC also remains unclear. Only a few cases with systemic chemotherapy for SGC were reported, such as 5−fluorouracil, doxorubicin, cyclophosphamide, and paclitaxel. In one report, a patient with bone and visceral metastases obtained complete response for 16 months after receiving chemotherapy consisting of doxorubicin, mitomycin C, vincristine, and cisplatin (15). Docetaxel has also been used in a patient with complete response for more than 2 years (16). However, no response could also be observed in some patients treated with 5−fluorouracil, doxorubicin, cyclophosphamide, or docetaxel (17, 18).

Immunotherapy has developed rapidly in recent years. It has already been applied in a variety of tumors such as melanoma, lung cancer, and gastric cancer. However, limited cases with immunotherapy have been reported in SGC. Two cases with metastatic porocarcinoma obtained good results after being treated with pembrolizumab, which is a PD-1 inhibitor (19, 20). One case reported by Lee et al. received complete response for 16 months after switching to pembrolizumab (19). The other case reported by Singh et al. showed partial remission for 18 months after the administration of pembrolizumab (20). Gupta reported that a patient who developed an eccrine carcinoma demonstrated a durable antitumor response after treatment with pembrolizumab (21). In this case, the patient survived for 21 months after initiation of camrelizumab. As seen above, immunotherapy may have the potential to become an alternative treatment for metastatic SGC.

In the case we reported here, the patient initially underwent local excision. Then, she received radiotherapy, multiple lines of chemotherapy, and immunotherapy, which consisted of docetaxel, nedaplatin, albumin-bound paclitaxel, pemetrexed, and camrelizumab. Although all the above treatment methods did not achieve significant benefit, they still helped slow down the progression of the tumor to some extent. Our case demonstrated that surgery combined with chemotherapy, radiotherapy, and immunotherapy is still an option to control SGC.

## Conclusion

In summary, SGC is a rare type of skin cancer with limited research. Surgical excision is the recommended treatment for a local SGC. There is no standard treatment for advanced SGC, and the outcomes are unclear. From this case report, chemotherapy with docetaxel, nedaplatin, albumin-bound paclitaxel, and pemetrexed, and immunotherapy with camrelizumab could be a suitable choice for advanced SGC.

## Data availability statement

The original contributions presented in the study are included in the article/supplementary material. Further inquiries can be directed to the corresponding author.

## Ethics statement

The studies involving human participants were reviewed and approved by Institutional Review Board of Tongji Hospital. The patients/participants provided their written informed consent to participate in this study.

## Author contributions

YD composed the manuscript and literature review. JF collected the case history and figures. XZ reviewed the manuscript. All authors contributed to the article and approved the submitted version.
